# Factors affecting do-not-resuscitate decisions among patients with amyotrophic lateral sclerosis in Taiwan

**DOI:** 10.1371/journal.pone.0282805

**Published:** 2023-03-13

**Authors:** Mei-Hsing Chuang, Jiunn-Rong Hsu, Chia-Wei Hung, Yu Long Hwang, Chih-Ching Lee, Hsiu-Yi Shen, Fu-Kang Chang, Li-Lin Kuo, Saint Shiou-Sheng Chen, Sheng-Jean Huang

**Affiliations:** 1 Division of Family Medicine, Taipei City Hospital Zhong Xiao Branch, Taipei, Taiwan; 2 Department of Family Medicine, School of Medicine, National Yang Ming Chiao Tung University, Taipei, Taiwan; 3 Division of Chest Medicine, Department of Internal Medicine, Taipei City Hospital Zhong Xiao Branch, Taipei, Taiwan; 4 Division of Neurology, Department of Internal Medicine, Taipei City Hospital Zhong Xiao Branch, Taipei, Taiwan; 5 Division of Palliative Medicine, Department of Internal Medicine, Taipei City Hospital Zhong Xiao Branch, Taipei, Taiwan; 6 Department of Nursing, Taipei City Hospital Zhong Xiao Branch, Taipei, Taiwan; 7 Department of Ophthalmology, Taipei City Hospital, Taipei, Taiwan; 8 Division of Urology, Taipei City Hospital Zhong Xiao Branch, Taipei, Taiwan; 9 Department of Urology, National Yang Ming Chiao Tung University, School of Medicine, Taipei, Taiwan; 10 Commission for General Education, College of Applied Science, National Taiwan University of Science and Technology, Taipei, Taiwan; 11 General Education Center, University of Taipei, Taipei, Taiwan; 12 Department of Surgery, College of Medicine, National Taiwan University, Taipei, Taiwan; National Center of Neurology and Psychiatry (NCNP), JAPAN

## Abstract

Amyotrophic lateral sclerosis (ALS) is a neurodegenerative disease. Usually, patients survive for approximately 2–4 years after the onset of the disease, and they often die of respiratory failure. This study examined the factors associated with signing a “do not resuscitate” (DNR) form in patients with ALS. This cross-sectional study included patients diagnosed with ALS between January 2015 and December 2019 in a Taipei City hospital. We recorded patients’ age at disease onset; sex; presence of diabetes mellitus, hypertension, cancer, or depression; use of invasive positive pressure ventilator (IPPV) or non-IPPV (NIPPV); use of nasogastric tube (NG) or percutaneous endoscopic gastrostomy (PEG) tube; follow-up years; and number of hospitalizations. Data from 162 patients were recorded (99 men). Fifty-six (34.6%) signed a DNR. Multivariate logistic regression analyses revealed that the factors associated with DNR included NIPPV (OR = 6.95, 95% CI = 2.21–21.84), PEG tube feeding (OR = 2.86, 95% CI = 1.13–7.24), NG tube feeding (OR = 5.75, 95% CI = 1.77–18.65), follow-up years (OR = 1.13, 95% CI = 1.02–1.26), and number of hospital admissions (OR = 1.26, 95% CI = 1.02–1.57). The findings suggest that end-of-life decision making among patients with ALS may often be delayed. DNR decisions should be discussed with patients and their families during the early stages of disease progression. Physicians are advised to discuss DNR with patients when they can speak and to offer palliative care options.

## Introduction

Amyotrophic lateral sclerosis (ALS)—a neurodegenerative disease—affects both the upper and lower motor neurons. The clinical symptoms and disease progression vary greatly [1]. The disease incidence and prevalence are approximately 0.78–2.35 and 3.01–7.96 per 100 000 people, respectively [2]. The onset of ALS peaks between 50–75 years [1]. Its etiology is unclear—gene mutation, environmental factors, viruses, toxin exposure, and autoimmunity may be related [1–3]. Patients may have symptoms of muscle weakness, dysphagia, and dyspnea, while muscles for eye movement and the sphincter remain unaffected [1]. Usually patients survive for approximately 2–4 years after the onset; they often die of respiratory failure [1–3].

The incidence of ALS in Taiwan is approximately 0.33–0.44 per 100 000 people; its prevalence is approximately 2.31 per 100 000 people [4]. In Taiwan, patients with ALS have an annual crude mortality rate of approximately 14.7%–19.7% within five years of diagnosis [5]. Average survival can be improved with riluzole, ventilators, or gastrostomy [4–6]. To date, the pathogenesis of ALS is not fully understood, and no effective treatment has been found [1–6]. Treatment includes symptomatic treatment and palliative care [7–9].

A Polish study of patients with ALS in the locked-in state found that some patients maintain a high sense of well-being despite severe physical restrictions [10]. Caring for patients with ALS is medically and financially resource intensive. Furthermore, there is a considerable burden on caregivers [11, 12]. Most patients with ALS are conscious and have normal sensory function. As the disease progresses, they become bedridden owing to immobility, have difficulty swallowing and breathing, and need to rely on tube feeding and a breathing ventilator. Patients with end-stage ALS require a tracheotomy to survive, and they often feel fatigued, depressed, pained, and hopeless—as if their souls are imprisoned in an immobile body.

Although many patients take life-sustaining measures, it is not uncommon for some to wish for death. According to surveys conducted in India and the US, 18.9–25% of patients said they wanted to die and 5.7% wanted to speed up their death [13–15]. A Dutch report stated that approximately 20% of patients with ALS chose euthanasia [16]. Reasons included fear of suffocation, feeling there is no chance for improvement, loss of dignity, dependence on others, and fatigue [16]. A US survey found that 71.4% (30 patients out of a sample of 42) of patients with ALS decided not to have cardiopulmonary resuscitation (CPR) administered [17].

Limited attempts have been made to identify the factors contributing to the signing of a DNR form in patients with ALS. Studies have reported that malnutrition, dementia, aspiration, very severe pneumonia, respiratory failure, albumin levels less than 3, Charlson Comorbidity Index higher than 2, and being transferred to the intensive care unit were independently associated with DNR orders among elderly people [18, 19]. In 2000, Taiwan promulgated the “Hospice Palliative Care Act” [20]. In this law, terminally ill patients refer to those who experience serious injuries or illness, have incurable diseases, or have medical evidence that shows that they have a fatal prognosis, in the near future. Additionally, this law stipulates that the terminal illness must be diagnosed by two physicians who are qualified and are specialists in the relevant field. If CPR is not to be performed, written consent is required from the patient or their closest relative, if the terminally ill patient has become unconscious or failed to clearly express his/her will [20]. CPR includes endotracheal intubation, chest compression, injection of resuscitation drugs, external defibrillation, artificial cardiac pacing, mouth-to-mouth ventilation, and ventilator use. Physicians provide palliative medical care to terminally ill patients according to their wishes [20]. The first ALS ward in Asia was established on October 15, 2006 at the Taipei City Hospital–Zhongxiao branch. A professional medical team took care of this group of patients. In the study hospital, the most common reasons for patients to be hospitalized were infectious diseases, dyspnea, and receiving percutaneous endoscopic gastrostomy (PEG). During hospitalization, various medical needs of patients are integrated by medical specialists. Patients may display improved quality of life and mental health. One study in Japan showed that communicating with patients and their families were important. This could help clinicians understand what patients require [21]. ALS does not have to be a terminal disease; however, when the condition is serious, the use resuscitation should coincide with their own wishes. This study examined the factors associated with signing a DNR form in patients with ALS.

## Materials and methods

### Sample

This cross-sectional study analyzed electronic inpatient and outpatient medical records of patients from a Taipei hospital between January 2015 and December 2019. Inclusion criteria were patients with an ALS diagnosis per the International Classification of Diseases (ICD) ninth revision (code 335.20) or ICD tenth revision (code G12.21). All patients were diagnosed by neurologists. The diagnoses were based on the revised El Escorial research diagnostic criteria for ALS [22]. Patients with ALS but not diagnosed by a neurologist were excluded.

Other factors recorded included basic information (onset age and sex); comorbidities (diabetes mellitus, hypertension, cancer, or depression); use of invasive positive pressure ventilator (IPPV) or non-IPPV (NIPPV); use of nasogastric tube (NG), PEG tube, or oral feeding; follow-up years; and number of hospitalizations since diagnosis. We also assessed whether a DNR form had been signed. This study was approved by the Human Research Ethics Review Committee of Taipei City Hospital (no: TCHIRB-10811001-E), and the need for informed consent was waived owing to the identification data of participants being removed before analysis.

### Statistical analysis

Statistical tests were performed using two-tailed tests, and *p*-values < .05 were considered significant. For descriptive statistics, chi-square and t-tests were conducted for categorical and numerical variables, respectively. For inferential statistics, a multivariable logistic regression analysis was conducted. Data analyses were performed using SAS 9.4 statistical software (SAS Institute, Inc., Cary, NC, USA).

## Results

**Fig 1:**
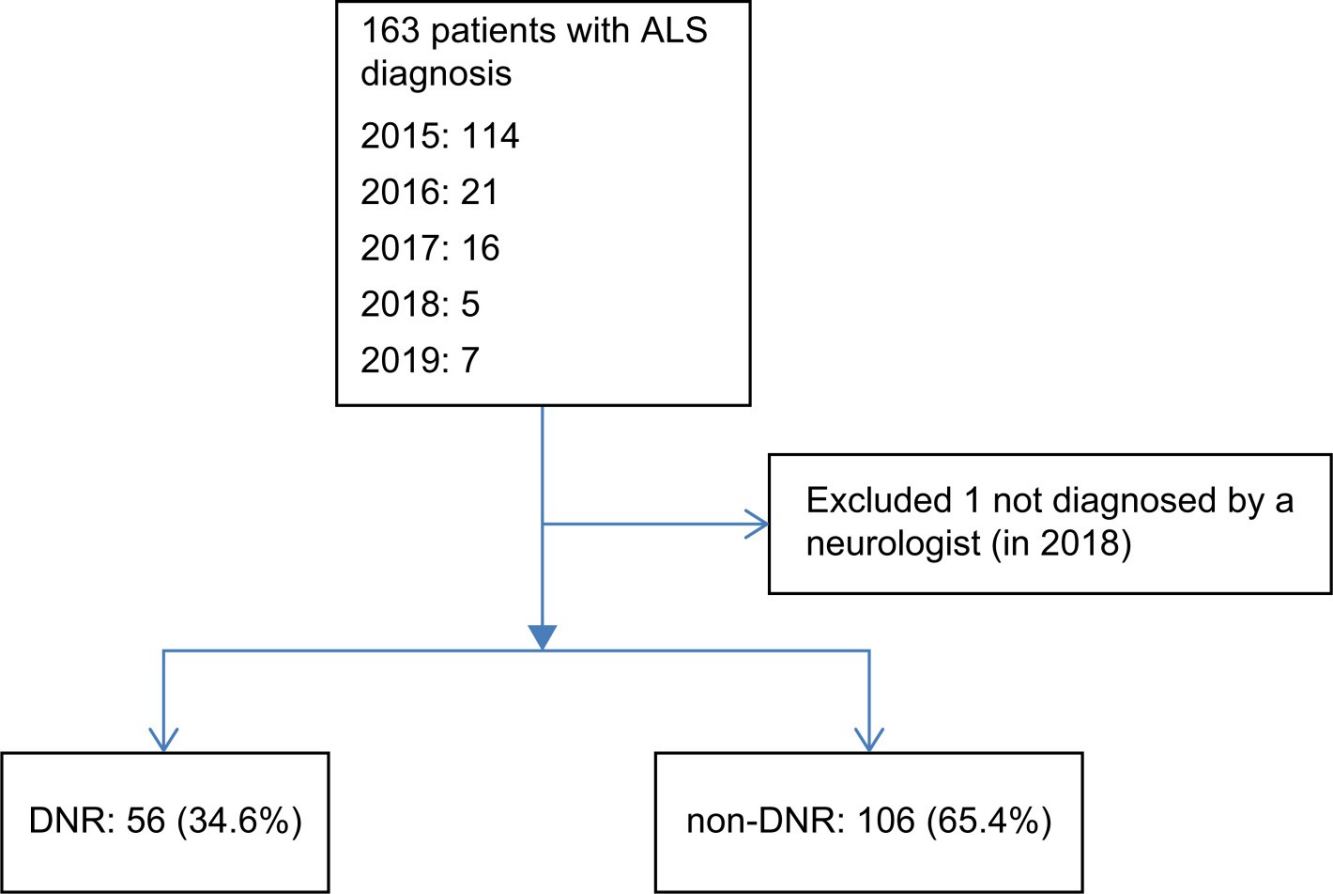
Process of case enrollment.

From January 2015 to December 2019, there were 163 patients. Of which, 162 patients with ALS diagnosed by neurologists were recorded (99 men, 61.1%) and one patient was excluded as they were not diagnosed by a neurologist (Fig 1). About one-third had signed a DNR form (DNR group), while nearly two-thirds had not (non-DNR group). Participants’ characteristics are shown in Table 1. The two groups were similar regarding the prevalence of hypertension, cancer, and depression; however, mean follow-up years, use of ventilator, tube feeding, prevalence of diabetes, and number of hospitalizations were higher in the DNR group compared to the non-DNR group. The average duration from onset to signing a DNR was 6.38 years (year of signing the DNR minus the year of disease onset).

**Table 1:** Characteristics of patients with amyotrophic lateral sclerosis by DNR^a^.

	No. (%) of patients			*p*
Characteristics	Total	DNR	non-DNR	
**n**	162 (100)	56 (34.6)	106 (65.4)	
**Demographics**				
**Sex**				
**Male**	99 (61.1)	33 (58.9)	66 (62.3)	.68
**Female**	63 (38.9)	23 (41.1)	40 (37.7)	
**Age of onset, years**				
**Mean ± SD**^b^	55.2 ± 12.5	57.6 ± 12.5	53.9 ± 12.4	.07
**25% Q1**	47	49.5	45	
**50% median**	57 (24–85)	59 (28–85)	53 (24–78)	
**75% Q3**	64	65	63	
**Follow-up years**				
**Mean ± SD**	5.9 ± 4.5	7.2 ± 5.0	5.3 ± 4.0	.01
**25% Q1**	2	3	2	
**50% median**	5 (1–20)	6 (1–20)	5 (1–20)	
**75% Q3**	8	10	7	
**Respiration**				
**IPPV**^c^	37 (22.8)	18 (32.1)	19 (17.9)	< .01
**NIPPV**^d^	65 (40.1)	32 (57.2)	33 (31.1)	
**No ventilator**	60 (37.1)	6 (10.7)	54 (51.0)	
**Feeding**				
**PEG**^e^	73 (45.1)	26 (46.4)	47 (44.3)	< .01
**NG**^f^	26 (16.1)	15 (26.8)	11 (10.4)	
**No tube feeding**	63 (38.8)	15 (26.8)	48 (45.3)	
**Comorbidity**				
**Diabetes mellitus**	31 (19.1)	20 (35.7)	11 (10.4)	< .01
**Hypertension**	51 (31.5)	23 (41.1)	28 (26.4)	.06
**Cancer**	4 (2.5)	3 (0.9)	1 (0.9)	.12
**Depression**	25 (15.4)	7 (12.3)	18 (17.0)	.45
**Number of hospitalizations**				
**Mean ± SD**	1.8 ± 2.3	3.0 ± 2.7	1.3 ± 2.1	< .01
**25% Q1**^g^	0	1	0	
**50% median**	1 (0–12)	2 (0–12)	0 (0–9)	
**75% Q3**	3	3	2	
**Mortality**	44 (27.2)	37 (66.0)	7 (6.6)	< .01

^a^DNR: do not resuscitate,

^b^SD: standard deviation,

^c^IPPV: invasive positive pressure ventilator,

^d^NIPPV: non-invasive positive pressure ventilator,

^e^PEG: percutaneous endoscopic gastrostomy,

^f^NG: nasogastric,

^g^Q: quartile.

We performed a collinearity analysis of all the variables; none of the univariate items were collinear. A univariate analysis of the factors associated with signing a DNR form in patients with ALS found that diabetes (*p* < .01), use of invasive (*p* = .01) or non-invasive ventilators (*p* = .003), use of NG tube feeding (*p* = .007), years of follow-up (*p* = .01), and number of hospitalizations (*p* < .01) were highly significant (Table 2). Variables that were significant in the univariate analysis were included in the multivariate analysis. A multivariate logistic regression analysis revealed that the factors associated with DNR included non-invasive ventilators, PEG tube feeding, NG tube feeding, follow-up years, and number of hospital admissions (Table 2).

**Table 2:** Univariate and multivariate analysis of factors associated with DNR^a^ among patients with amyotrophic lateral sclerosis.

	Univariate analysis	Multivariate analysis
Variables	OR^b^ (95% CI)	AOR^c^ (95% CI)
**Sex**		
**Male**	0.87 (0.45–1.69)	0.80 (0.34–1.88)
**Female**	1	1
**Age of onset, years**	1.03 (0.99–1.05)	1.03 (0.99–1.07)
**Follow-up years**	1.10 (1.02–1.18)*	1.13 (1.02–1.26)*
**Respiration**		
**IPPV**^d^	8.53 (2.95–26.65)*	2.91 (0.69–12.24)
**NIPPV**^e^	8.73 (3.30–23.10)*	6.95 (2.21–21.84)*
**No ventilator**	1	1
**Feeding tube**		
**PEG** ^f^	1.77 (0.83–3.76)	2.86 (1.13–7.24)*
**NG**^g^	4.36 (1.65–11.51)*	5.75 (1.77–18.65)*
**No tube feeding**	1	1
**Comorbidity**		
**Diabetes mellitus**	4.80 (2.09–11.00)*	2.65 (0.93–7.51)
**Hypertension**	1.94 (0.98–3.85)	
**Cancer**	5.94 (0.60–58.46)	
**Depression**	0.70 (0.27–1.79)	
**Number of hospitalizations**	1.49 (1.23–1.80)*	1.26 (1.02–1.57)*

^a^DNR: do not resuscitate,

^b^OR: odds ratio,

^c^AOR: adjusted odds ratio,

^d^IPPV: invasive positive pressure ventilator,

^e^NIPPV: non-invasive positive pressure ventilator,

^f^PEG: percutaneous endoscopic gastrostomy,

^g^NG: nasogastric.

* Significant.

## Discussion

Fifty-six people (34.6%) had signed a DNR form (DNR group). This contradicts an American study in which 71.4% (30 patients out of a sample of 42) had decided on a DNR, regardless of their level of respiratory insufficiency [17]. Furthermore, the related factors for the DNR-group patients in this study differed. We found that NG or PEG tube, number of hospitalizations, use of ventilator, and years of follow-up increased the probability of signing a DNR. Further, NG and PEG tubes were significant in the multivariate analysis. This is consistent with physicians’ clinical experience. When a patient has dysphagia and needs a tube feeding diet, they may consider signing a DNR form. However, IPPV was not significant in the multivariate analysis. The 95% CI is relatively wide, which we speculate to be because of the small sample size. Doctors’ clinical experience allows them to know when a patient has difficulty breathing despite using NIPPV, and therefore, requires IPPV, they may consider signing a DNR form. The reason for the difference may be that physicians in the US discuss end-of-life decisions with patients during their first visit [17], while discussing death is still taboo in Taiwan. Compared to other diseases in Taiwan, a study reported that 19.6% (66 out of 337) of the geriatric patients signed a DNR form during hospitalization [19].

Most patients with ALS signed a DNR when the disease was more severe. The average duration from onset to signing a DNR was 6.38 years. This hospital provided a professional medical team to take acute care of ALS patients; thus, patients’ survival period was relatively long. The study hospital also has a respiratory care unit for those who must rely on ventilators for a long time. Another reason was that Taiwan’s national health insurance covers all outpatient, inpatient, and hospice care for patients with ALS [23, 24]. Therefore, patients with ALS or their family usually decide whether to sign a DNR at the last minute.

Another reason may be that physicians do not inform patients of the need for a tracheostomy for future respiratory failure during the initial diagnosis [25, 26]. Until now, there is still no satisfactory treatment for ALS. For such patients, palliative care may be an appropriate treatment choice [7, 27, 28]. According to the experience of our doctors who care for ALS patients, after being judged as terminally ill by two doctors, the following two situations can be regarded as futile medical care: the first is when a patient has progressed from NIPPV to requiring IPPV, and the second is when the patient is dying and he is already on an IPPV. Both of these situations are extremely difficult moments for patients or their families to decide during. If the patient does not pre-declare not to do so, the physician will perform endotracheal intubation or CPR. Hospice palliative care is supportive medical care that is provided to help relieve terminally ill patients from physical, mental, and spiritual pain. Hospice training can help doctors discuss end-of-life decisions and future treatment plans [29, 30]. Patients with ALS may sustain cognitive impairments with disease progression [31–34], such as frontotemporal lobar degeneration and frontotemporal dementia [1]. Early discussion of end-of-life decisions can avoid delays owing to an unpredictable disease progression [17]. Therefore, after diagnosis, integrating advance care planning into the follow-up is recommended [28].

Taiwan’s National Health Insurance Bureau implemented the registration of the “DNR Willing Form” into the health insurance integrated circuit (IC) card. Hence, a physician can read patients’ “no CPR” on the IC card [35]. The most humane mode of care for terminally ill patients with ALS is palliative care. In this study, only one-third of ALS patients in the hospital signed a DNR form, and more than one-fifth of them used IPPV. It is suggested that doctors should discuss palliative care with patients at the right time so that patients are more comfortable and suffer less. Physicians and family members can assist the passing of the patient in a peaceful manner according to their wishes.

This study has several strengths, including providing insights into the association of patients’ functional status in their DNR decision making and considering several comorbidities to adjust for multiple confounding variables. Furthermore, patients were followed for up to five years. However, this study also has some limitations. First, we failed to consider patients’ and family members’ religion, educational level, socioeconomic status, serum albumin levels, and cognitive factors. Second, participants were limited to patients from only one hospital, which limits the generalizability of our results to all patient with ALS.

## Conclusion

The factors associated with DNR in patients with ALS included non-invasive ventilators, PEG tube feeding, NG tube feeding, follow-up years, and the number of hospital admissions. These factors all appeared in the late stage of the disease, suggesting delayed DNR decision making in patients with ALS. DNR decisions should be discussed with patients and their families during disease progression. We suggest physicians assist them in making appropriate care decisions, avoid futile medical care, and maintain patients’ end-of-life dignity. Furthermore, a referral to specialist palliative care is encouraged to improve patients’ quality of life.

## Supporting information

S1 Checklist STROBE statement—Checklist of items that should be included in reports of observational studies. (DOCX)
